# Integrating Nutrition into Precision Medicine for Controlling Systemic Inflammation in Rheumatoid Arthritis

**DOI:** 10.1007/s10753-025-02373-7

**Published:** 2025-12-17

**Authors:** Francesca Ingegnoli, Saviana Gandolfo, Francesco Ciccia, Roberto Caporali

**Affiliations:** 1 Rheumatology Clinic, Department of Rheumatology and Medical Sciences, ASST Gaetano Pini-CTO, Milan, Italy; 2 Department of Clinical Sciences and Community Health, Dipartimento di Eccellenza 2023–2027, Università degli Studi di Milano, Milan, Italy; 3 Rheumatology Section, Department of Precision Medicine, University della Campania L. Vanvitelli, Naples, Italy; 4 Rheumatology Unit, Ospedale del mare, Naples, Italy

**Keywords:** Nutrition, Rheumatoid arthritis, Precision medicine, Inflammation

## Abstract

The management of rheumatoid arthritis (RA) has evolved from the conventional “treat-to-target” approach to strategies that emphasize individualized, patient-centered care. Concepts such as “therapeutic matchmaking” and “smart-to-target” highlight the importance of biomarkers, comorbidities, and quality-of-life priorities in selecting treatments. There is increasing evidence that identifies the gut-joint axis as a critical factor in RA pathogenesis. This axis links gut microbiota composition, intestinal barrier integrity, and systemic inflammation. Dysbiosis, reduced short-chain fatty acid production, and increased gut permeability can lead to immune dysregulation and joint inflammation. Nutritional interventions, such as high-fiber diets, prebiotics, probiotics, polyphenols, and omega-3 fatty acids, can restore microbial balance, enhance barrier function, and reduce inflammation. Precision nutrition, which tailors dietary recommendations based on genetic, microbiome, metabolic, and lifestyle factors, provides a framework for incorporating gut health into RA management. Microbiome-guided dietary strategies may improve responses to pharmacologic treatments, enable early intervention, and provide preventive benefits for high-risk individuals. Combining personalized nutrition with pharmacotherapy could optimize disease control, mitigate side effects, and promote sustainable, patient-empowering care. Future research should focus on randomized controlled trials and advanced analytic tools to refine predictive models and establish personalized nutrition as a cornerstone of holistic RA management.

## Introduction

The management of systemic inflammation in rheumatoid arthritis (RA) remains a complex challenge in rheumatology, despite the multitude of therapeutic strategies that have emerged over the years to treat this complex and heterogeneous disease. The “treat-to-target” (T2T) approach has dominated clinical practice for years, advocating the achievement of well-defined levels of disease activity, such as remission or low disease activity (LDA), through continuous modulation of therapy [1]. Recently, the field has turned to the possibility of precision medicine, the idea of using biomarkers and patient-specific profiles, including comorbidities and drug safety profiles, to identify the best drug for the best patient [2]. Another interesting strategy could be a “smart-to-target” approach through the definition of more realistic and patient-centered therapeutic goals. By combining the consolidated “treat to target” approach with a greater attention to health-related quality of life aspects such as nutrition, this strategy could offer a more holistic therapeutic approach than simply adhering to rigid disease activity benchmarks [2]. Indeed, the progression of RA treatment cannot fail to involve a more comprehensive and integrative approach, which does not forget fundamental pieces of the puzzle, including often overlooked environmental and lifestyle factors, smoking aside. It is obvious that, unlike conventional pharmacological interventions with clearly defined therapeutic targets, the role of diet is much more difficult to quantify. Personal preferences, seasonal and territorial food availability, economic impact and socio-cultural aspects add further layers of complexity, often relegating attention to nutritional aspects to the background. This is reflected in generic recommendations such as those present in the recommendations of the European Alliance of Associations for Rheumatology (EULAR) and the American College of Rheumatology (ACR) [3–5].

### The Gut-Joint Connection: A Nutritional Frontier

The interaction between the gut and joints, known as the gut-joint axis, has emerged as an exciting area of research for understanding the pathophysiology and potential therapeutic management of RA [6, 7]. This connection highlights how gut health profoundly influences systemic inflammation, which is central to the pathophysiology of RA [6, 7]. Nutrition appears to play a fundamental role in shaping this axis, primarily through its impact on the gut microbiota and intestinal barrier function [8, 9]. The human gut microbiome consists of trillions of microorganisms that contribute to immune modulation, body metabolism, and the maintenance of normal intestinal barrier function [10]. In RA, dysbiosis, an imbalance in the composition and function of the gut microbiota compared to controls, has been linked to both the early stages of joint inflammation and its perpetuation [6, 7]. This imbalance often leads to a reduction in beneficial microbial species and an overgrowth of microbes with pro-inflammatory properties. In such a profoundly altered ecosystem, the intestinal barrier, a key defense mechanism in the separation between the intestinal lumen and immune cells of the lamina propria, can become “permeable”, allowing microbial products, such as lipopolysaccharide (LPS), to translocate into the lamina propria, activating innate and adaptive immune cells and then into the bloodstream, creating a systemic pro-inflammatory milieu [7]. These microbial products can indeed activate the immune system, promote low-grade systemic inflammation and trigger autoimmune responses that can affect the joints [6, 7].

### Nutritional Strategies to Restore Gut Homeostasis

Dietary interventions may play a key role in modulating the altered gut microbiome, restoring proper epithelial barrier function, and positively modulating the gut-joint axis balance [11]. Dietary fiber, particularly from plant-based foods, is fermented by gut bacteria to produce short-chain fatty acids (SCFAs) such as butyrate, propionate, and acetate. SCFAs have profound anti-inflammatory effects, promoting intestinal barrier integrity and reducing inflammation at both the intestinal and systemic levels [12]. Studies in mouse models of RA have shown that increasing SCFA levels through high-fiber diets or direct supplements can alleviate joint inflammation and prevent disease progression [13–15]. In this scenario, probiotics, live beneficial microbes, and prebiotics, non-digestible fibers that nourish these microbes, can be used to selectively increase the abundance of anti-inflammatory gut bacteria [16]. For example, Bacteroides fragilis, which produces the SCFA propionate, has been identified as a critical factor in reducing RA symptoms by modulating the pro-inflammatory phenotype of fibroblast-like synoviocytes (FLS) [17]. Including prebiotic-rich foods such as garlic, onions, and bananas in the diet may promote the growth of such beneficial strains [18, 19]. Foods rich in polyphenols, such as berries, green tea, and olive oil, have antioxidant and anti-inflammatory properties that may contribute beneficially [20, 21]. Polyphenols, in fact, also support the growth of beneficial gut bacteria, such as Lactobacillus and Bifidobacterium, which are often reduced in RA patients. These bioactive compounds may help modulate both intestinal and systemic inflammation [22]. Omega-3 fatty acids, found in fatty fish and flaxseed, have also been shown to exert anti-inflammatory effects by reducing pro-inflammatory cytokines [23]. These fats also support intestinal health by promoting microbial diversity, an important factor for maintaining a healthy microbiome and an intact intestinal barrier [23]. Such dietary interventions, although always recommended in patients with RA, regardless of the early or late phase of the disease, could be fundamental in the pre-clinical phases of the disease, i.e. in patients with inflammatory joint pain and autoantibody positivity.

### Gut Barrier Integrity: A Target for RA Management

The intestinal epithelial barrier acts as a gatekeeper, preventing the translocation of harmful microbes and their products into the lamina propria and systemic circulation, while allowing nutrient absorption [24]. In RA, the integrity of the intestinal barrier is compromised and is associated with increased local immune activation and systemic inflammation [6]. Nutritional interventions aimed at restoring the integrity of this barrier could provide significant therapeutic benefits [25]. Zonulin, a protein involved in the modulation of intestinal permeability, plays a crucial role in inducing increased intestinal permeability through the disassembly of tight junctions. Elevated zonulin levels have been linked to increased intestinal permeability in patients with RA and pre-RA [26]. Serum zonulin concentrations have also been shown to predict, with good sensitivity and specificity, the transition from pre-RA to full-blown RA. Diets rich in plant fiber and SCFA-producing bacteria are able to modulate zonulin activity, restoring barrier function and reducing inflammation [27]. Supplementation with butyrate or propionate has shown promise in restoring intestinal barrier function in preclinical models [17, 28]. These SCFAs enhance tight junctions between epithelial cells, preventing microbial translocation and dampening intestinal and systemic immune activation [29]. Fecal microbiota transplantation (FMT) has emerged as a potential strategy to restore a healthy microbiome in RA [17]. A recent study demonstrated that transplantation of microbiota from arthritis-resistant mice to susceptible mice conferred resistance to the development of arthritis [17]. Researchers identified Bacteroides fragilis as a key factor contributing to this effect, underscoring the therapeutic potential of microbiota modulation [17]. Although human studies are still in their infancy, FMT may represent a novel, non-pharmacological approach to RA management in the future.

The gut microbiome not only influences the pathogenesis of RA but also influences responses to pharmacological treatments [30]. For example, differences in gut microbial composition have been associated with different responses to methotrexate, the first-line treatment for RA [31–33]. Microbiome profiling may help identify RA patients who may benefit from different pharmacological therapies, paving the way for precision medicine also guided by the gut microbiome [34]. Furthermore, dietary strategies that increase the abundance of beneficial microbes may act synergistically with pharmacological treatments, improving their efficacy and reducing side effects [35]. For RA patients seeking practical dietary advice, the importance of whole, minimally processed foods that support gut health should be emphasized [11]. Encouraging the consumption of fiber-rich fruits and vegetables, fermented foods such as yogurt, and foods rich in anti-inflammatory fats may enable patients to take a truly active role in managing their condition [36, 37]. At the same time, limiting pro-inflammatory foods, such as refined sugars, saturated fats, and highly processed foods, may reduce dysbiosis and systemic inflammation [9]. By integrating dietary strategies with microbiome-targeted therapies, clinicians can offer patients a more holistic approach to disease management [38]. These interventions, in fact, could address the root causes of inflammation in RA, in line with the principles of precision nutrition, through dietary personalization based on each patient’s biological context and lifestyle. In the future, advances in understanding the microbiome and the complex interconnections between the microbiome and the immune system could reshape the standard of care for RA, offering sustainable, non-invasive, and patient-centered solutions. In this scenario, the gut-joint connection would no longer be a marginal and, for many, speculative consideration, but could represent a central pillar in the understanding and management of RA.

### Precision Nutrition: A Personalized Approach to RA Management

**Fig. 1:**
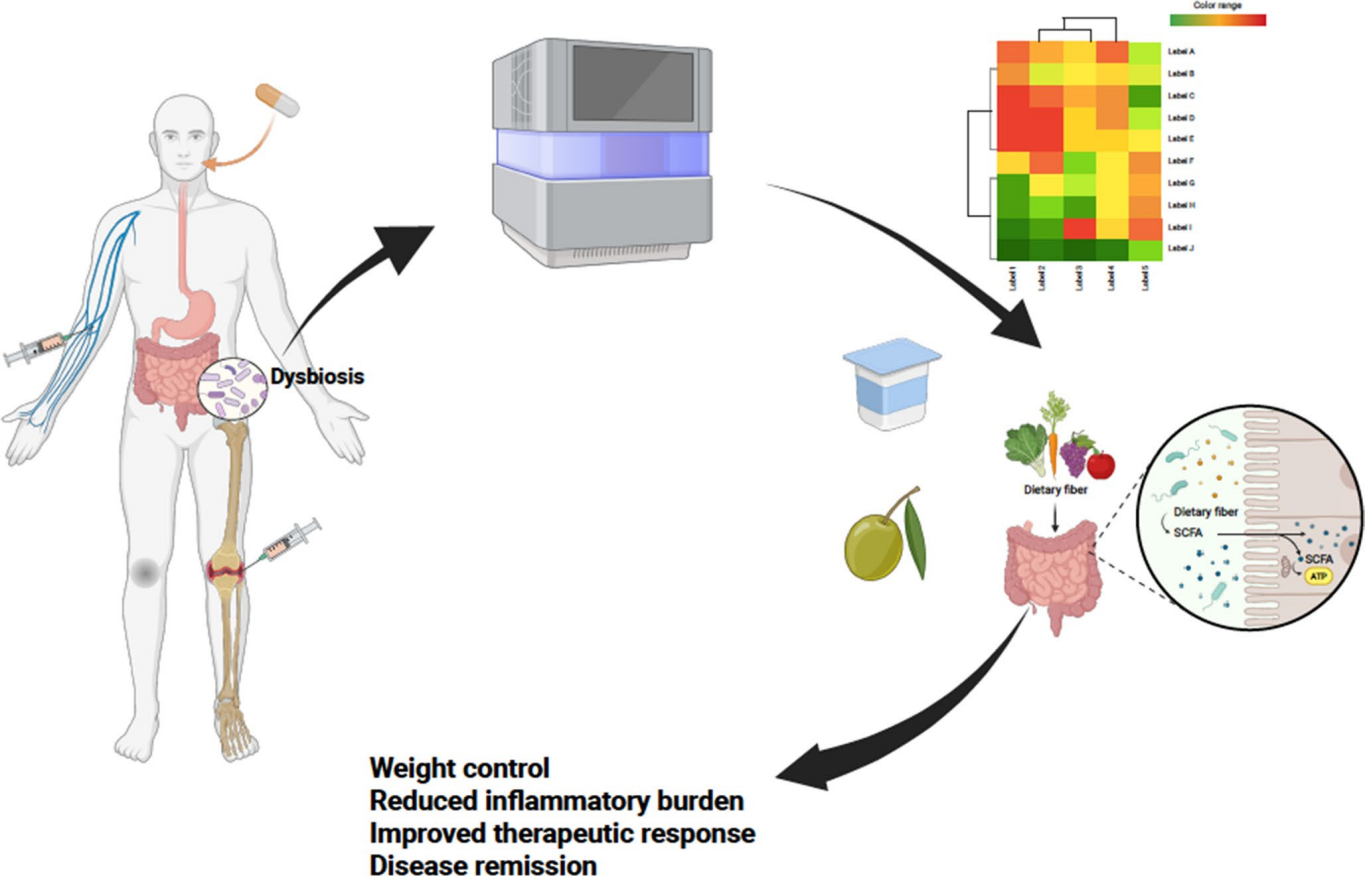
Conceptual framework for microbiome-guided dietary interventions in rheumatoid arthritis (RA). Schematic representation of a precision medicine approach integrating intestinal microbiome profiling in patients with RA. Analysis of gut microbial composition may enable the identification of individuals with dysbiosis, who may benefit from targeted nutritional interventions aimed at restoring microbial balance. Such dietary modulation is proposed as an adjunct to standard pharmacological therapy to reduce systemic inflammation and disease activity. Created in BioRender. Ciccia, F. (2025) https://BioRender.com/9jut4wv.

With the increasing shift in medicine towards personalized care, the concept of precision medicine, which also includes the possibility of precision nutrition, has emerged as a promising frontier in the management of complex diseases such as RA [39]. Precision nutrition is an innovative concept that tailors nutritional recommendations to the unique biological, environmental and lifestyle characteristics of an individual [40]. By integrating data from genetics, epigenetics, microbiome composition analysis, metabolic profiles, global health status and personal behaviors, precision nutrition attempts to prevent the onset of pathologies (e.g. metabolic diseases) and improve responses to conventional treatments (Fig. 1) [40]. Unlike generalized dietary guidelines, precision nutrition indeed tries to take into account interindividual variability in nutrient metabolic processes, physiological or pathophysiological responses to food, aiming for nutritional strategies that are as personalized as possible and at the same time effective and sustainable. This ongoing paradigm finds room for exploration in inflammatory bowel disease (IBD), where the long-term goal is to try to answer the key question of patients affected by IBD: “what should I eat?” [41]. A similar approach could also be applied to RA, addressing not only systemic inflammation, but also the underlying gut-joint connection that plays a critical role in disease activity and progression [6]. Evidence supports the role of anti-inflammatory diets, such as the Mediterranean diet, in reducing RA-related inflammation [42–44]. However, a one-size-fits-all approach often fails to address individual variability in responses to dietary interventions [43]. Precision nutrition should also consider factors such as gut microbiota composition, genetic predispositions, and metabolic profiles. For example, the high fiber content of plant-based diets promotes the production of SCFAs, such as butyrate and propionate, which are known to strengthen the intestinal barrier and reduce systemic inflammation [45]. In precision nutrition, gut microbiota analysis could identify patients lacking specific SCFA-producing bacterial strains, allowing for targeted interventions through diet or pre/probiotic supplementation [46]. Similarly, omega-3 fatty acids, found in oily fish, have demonstrated anti-inflammatory effects, but genetic variations in fatty acid metabolism could influence an individual’s response, necessitating personalized dietary plans [23].

### Microbiome-Driven Dietary Strategies

The gut microbiome appears to play a critical role in the pathogenesis and progression of RA [6]. Precision nutrition could leverage gut microbiome profiling to design diets that, by reshaping microbial communities, could ultimately reduce local and systemic inflammation, potentially even improving treatment outcomes. Studies have shown that dysbiosis, characterized by a decrease in beneficial species and an increase in pro-inflammatory species, may contribute to RA symptoms. Addressing these imbalances through personalized dietary interventions could represent a very promising strategy [6, 47].

For example, patients with low levels of Bacteroides fragilis, a bacterium known to produce the SCFA propionate, may benefit from diets rich in plant fiber or the use of probiotics containing B. fragilis [17, 48]. This approach could not only improve gut health but also enhance the efficacy of existing pharmacological treatments, as demonstrated in studies where B. fragilis supplementation improved responses to anti-TNF therapy in mouse models [17, 49]. By combining dietary strategies with microbiome modulation, precision nutrition may offer a complementary route to achieve control of systemic inflammation.

### Addressing Metabolic and Immune Variability

RA is characterized by marked heterogeneity in patients’ immune response and metabolic activity, factors that can significantly influence both dietary needs and therapeutic responses. It should be acknowledged, however, that accurately titrating the composition and concentration of individual dietary components is extremely challenging, if not impossible, in the context of a personalized diet, whereas such precision is achievable with standardized probiotic supplementation, thus supporting the distinction between personalized nutrition and precision nutrition. In this regard, personalized nutrition could enable the creation of personalized diets designed based on an individual’s immune and metabolic profile (Fig. 1). For example, low-protein diets are able to suppress FLS-mediated pro-inflammatory pathways [50], inhibit the polarization of pro-inflammatory M1 macrophages, and promote the M2 anti-inflammatory phenotype through the activation of the NRF2/SIRT3/SOD2 pathway [51]. Precision nutrition could identify RA patients whose metabolic profiles suggest sensitivity to protein intake and recommend personalized diets to balance protein levels while maintaining overall nutritional adequacy. Similarly, sodium and magnesium intake, which modulate immune responses, could be optimized based on an individual’s baseline electrolyte levels and immune activity [52, 53]. For example, patients with a propensity for elevated Th17-driven inflammation may benefit from reduced magnesium and salt intake to attenuate Th17-dependent pro-inflammatory pathways [53, 54].

### Precision Nutrition as an Adjunct to Pharmacotherapy and for Prevention and Early Intervention in RA

One of the most exciting aspects of precision nutrition is its potential to improve the efficacy of drug treatments. Studies have shown that the composition of the gut microbiome can influence responses to drugs such as methotrexate and anti-TNFα drugs [17, 31, 49]. On the other hand, recent evidence suggests that DMARDs, including biologics such as etanercept, may partially restore gut microbiota composition and metabolic balance in rheumatoid arthritis, likely through the reduction of systemic inflammation and disease activity [55, 56]. Patients with specific microbiome characteristics may achieve better therapeutic outcomes with dietary strategies specifically integrated into their treatment regimens. For example, patients with a microbiome rich in SCFA-producing bacteria may show improved responses to certain biologics [57, 58]. Precision nutrition could support these patients by recommending diets that support and promote these beneficial microbial populations. Conversely, patients with dysbiosis may require targeted dietary interventions to restore the gut microbiome before initiating drug therapy, potentially improving both drug efficacy and reducing side effects [59]. As previously mentioned, in addition to improving management of established disease, precision nutrition could represent a promising strategy for RA prevention and to improve early therapeutic intervention approaches. By identifying individuals at high risk of RA based on their genetic predisposition and microbiome profile, precision nutrition could offer preventive strategies to reduce the likelihood of disease onset [60]. Diets enriched with anti-inflammatory nutrients, combined with targeted microbiome modulation, could serve as preventive measures for individuals with early signs of systemic and/or autoimmune inflammation. In patients with early RA, precision nutrition can be used to mitigate disease progression by addressing metabolic dysfunctions and reducing oxidative stress [61]. For example, diets rich in antioxidants, such as polyphenols found in berries and green tea, could help counteract oxidative damage in susceptible individuals. Individualized dietary plans can also focus on reducing key “metabolic” inflammatory factors, such as saturated fats and refined sugars, while promoting nutrient-dense, anti-inflammatory foods. This approach may positively impact both disease activity and comorbidities [62, 63].

### The Future of Precision Nutrition in RA

To fully realize the potential of precision nutrition in RA, rigorous research is needed. Randomized, controlled clinical trials will explore the impact of personalized dietary interventions on disease activity, microbiome composition, and treatment outcomes. The integration of advanced technologies, such as metabolomics combined with machine learning, will likely play a crucial role in developing predictive models that guide the most personalized dietary recommendations possible. Looking ahead, precision nutrition has the potential to become a cornerstone of RA management, complementing pharmacological, dietary, and lifestyle treatments. By integrating personalized dietary strategies with established therapies, clinicians can offer RA patients a holistic, yet accessible and sustainable approach to improving their quality of life.

## Data Availability

No datasets were generated or analyzed during the current study.
